# Supplementary material for: Higher ratio of plasma omega-6/omega-3 fatty acids is associated with greater risk of all-cause, cancer, and cardiovascular mortality: A population-based cohort study in UK Biobank
Source: eLife. 2024 Apr 5;12:RP90132. doi: 10.7554/eLife.90132 (PMC10997328; doi:10.7554/eLife.90132)
Supplement: Supplementary file 1. [file elife-90132-supp1.docx]

| **Table S1**. Literature Review | | | | | | | | | |
| --- | --- | --- | --- | --- | --- | --- | --- | --- | --- |
| **Author** | **Year** | **Study design/ location** | **Endpoints** | **# Cohort** | **# Cases** | **Exact exposure** | **Exposure characterization** | **Measure of associations** | **P for trend** |
| *Omega-6/omega-3 ratio* | | | | | | | | | |
| Noori et al.^1^ | 2011 | Prospective cohort/ hemodialysis patients, Southern California during 2001-2007 | All-cause mortality | 145 | 42 | Dietary Omega-3  Dietary Omega-6/3 | HR in quartiles (95% CI) | (Highest to lowest) 0.65 (0.21-1.97)  (Lowest to highest) 0.37 (0.14-1.08) | 0.1  0.04 |
| Harris et al.^2^ | 2017 | Prospective cohort/ women, Women’s Health Initiative Memory Study, enrollment began 1996, age 65-80 | All-cause mortality  CVD mortality  Cancer mortality | 6,501 | 1,851  617  462 | Red blood cell  Omega-3 index (EPA + DHA)  EPA  DHA  LA  N-6/N-3 ratio | HR per 1-SD increase in red blood cell PUFA (99% CI) | All  Omega-3 index: 0.92 (0.85-0.98)  EPA: 0.89 (0.82-0.96)  DHA: 0.93 (0.87-1.00)  LA: 0.99 (0.93-1.06)  N-6/N-3 ratio: 1.10 (1.02-1.19)  CVD  Omega-3 index: 0.97 (0.85-1.12)  EPA: 0.88 (0.77-1.00)  DHA: 1 (0.87-1.14)  LA: 0.96 (0.86-1.08)  N-6/N-3 ratio: 1.05 (0.9-1.23)  Cancer  Omega-3 index: 0.92 (0.79-1.07)  EPA: 0.91 (0.78-1.07)  DHA: 0.93 (0.79-1.09)  LA: 0.94 (0.83-1.07)  N-6/N-3 ratio: 1.1 (0.93-1.3) | 0.0015  < 0.001  0.0099  0.8286  0.0021  NR  NR |
| Otsuka et al.^3^ | 2019 | Prospective cohort study/ NILS-LSA, elderly individuals, Japan | All-cause mortality | 1054 | 422 | n-6 intake  n-3 intake  EPA intake  DHA intake  Serum EPA  Serum DHA  Serum EPA/ARA | HR in tertiles (Highest to lowest; 95% CI) | 0.80 (0.59-1.07)  0.83 (0.61-1.12)  0.96 (0.71-1.30)  0.95 (0.70-1.28)  0.81 (0.60-1.09)  0.73 (0.53-0.99)  0.71 (0.53-0.96) | 0.13  0.22  0.78  0.72  0.17  0.047  0.02 |
| Zhuang et al.^4^ | 2019 | Population based prospective cohort/ CHNS in China and NHANES in US | All-cause mortality | CHNS: 14,117  NHANES: 36,032 | 1,007  4,826 | Total PUFA intake  Omega-3 intake  Omega-6 intake  Omega 6/3 ratio | HR in quartiles (Highest to lowest; 95% CI) | China: 1.19 (0.93-1.52)  US: 0.86 (0.71-1.03)  China: 1.22 (1.00-1.50)  US: 0.85 (0.71-1.01)  China: 1.14 (0.89-1.47)  US: 0.84 (0.70-1.01)  China: 0.95 (0.80-1.14)  US: 0.99 (0.89-1.11) | 0.10  0.05  0.05  0.03  0.29  0.04  0.54  0.85 |
| *Circulating PUFAs* | | | | | | | | | |
| Wang et al.^5^ | 2003 | An epidemiological survey of 65 rural countries in China | All-cause mortality | 6500 | NR | DHA  EPA  Total plasma Omega-3  Total plasma Omega-6 | Pearson correlation coefficient | Around -0.3 (mortality)  Around -0.2 (mortality)  -0.152  -0.001 | NR |
| Lindberg et al.^6^ | 2008 | Prospective cohort/ Norway, elderly patients (mean=82.1), 3 y of follow-up | All-cause mortality | 254 | 101 | Plasma EPA | HR for quantile 1 (lowest, ref; 95% CI) compared with upper 3 quartiles combined | 0.52 (0.35-0.77) | NR |
| Chattipakorn et al.^7^ | 2009 | Measure the heart tissues of cadavers with a history of CHD | - | 100 cadavers | - | Omega-3 (EPA + DHA)  Omega-6 (AA + LA) | P-value from two-sample Wilcoxon rank sum test (cardiac cause vs. noncardiac cause.  Lower in the group with cardiac mortality) | Cadavers with heart disease  0.040  0.022 | NR |
| Lee et al.^8^ | 2009 | Prospective cohort/ patients with acute myocardial infarction, 16.1 months follow-up | All-cause mortality | 508 | 36 | Plasma EPA | - | Lower plasma level of EPA was and independent predictor for all-cause mortality in female patients | NR |
| Pottala et al.^9^ | 2010 | Prospective cohort/ outpatients with stable coronary heart disease, recruited between 2000-2002, San Francisco Bay Area | All-cause mortality | 956 | 237 | EPA + DHA in whole blood | HR above vs. below median (95% CI) | 0.74 (0.55-1.00) | 0.049 |
| Petrone et al.^10^ | 2012 | Nested case-control/ US male physicians | Heart failure | 788 cases  788 matched controls | - | Plasma omega-6 PUFAs | OR in quartiles (Highest to lowest; 95% CI) | 0.87 (0.63-1.20) | 0.39 |
| Mozaffarian et al.^11^ | 2013 | Prospective cohort/ older adults not taking fish oil supplements, 1992-2008, four US communities | All-cause mortality  CVD mortality | 2,692 | 1,625  570 | Plasma Total n-3 PUFA | HR in quintiles (Highest to lowest; 95% CI) | 0.73 (0.61-0.86)  0.65 (0.48-0.87) | <0.001  < 0.001 |
| Wu et al.^12^ | 2014 | Community-based prospective cohort/ Cardiovascular Health Study, age>=65, US | All-cause mortality  CVD mortality | 2,792 | 1,994  678 | Plasma phospholipid n-6 PUFA  LA  (Others are NS) | HR in quintiles (Highest to lowest; 95% CI) | All-cause mortality  0.87 (0.74-1.02)  CVD mortality  0.78 (0.60-1.01) | 0.005  0.02 |
| Eide et al.^13^ | 2016 | Cross-sectional study/ Renal transplant recipients, transplanted 1999-2011 | CVD mortality | 1990 | NR | plasma Marine n-3 PUFA levels | Per 1.0 wt% increase (95% CI) | 0.90 (0.82-0.98) | NR |
| Kleber et al.^14^ | 2016 | Prospective cohort/ patients referred for coronary angiography, LURIC study, German | All-cause mortality  CVD mortality | 3259 | 975  614 | erythrocytes (red cells)  EPA  HS-Omega-3 Index | HR in tertiles (Highest to lowest; 95% CI) | EPA  All: 0.75 (0.64-0.88)  CVD: 0.70 (0.57-0.86)  HS-Omega-3 Index  All: 0.78 (0.67-0.92)  CVD: 0.78 (0.64-0.95) | 0.001  0.003  0.009  0.050 |
| Miura et al.^15^ | 2016 | Population based prospective cohort/ Austria, drawn from Nambour Skin Cancer Study, in 1986 | All-cause mortality | 1,008 | 179  M: 98  F: 81 | Plasma total Omega-3  EPA  DPA  DHA  Total Omega-6 | HR per 1-SD increase (95% CI) | All: 0.96 (0.82-1.12)  M: 0.99 (0.80-1.22) F: 0.95 (0.76-1.19)  All: 0.81 (0.69-0.95)  M: 0.78 (0.62-0.98) F: 0.78 (0.65-0.94)  All: 0.90 (0.77-1.05)  M: 0.76 (0.60-0.97) F: 0.98 (0.79-1.22)  All: 1.07 (0.92-1.25)  M: 1.12 (0.92-1.36) F: 1.02 (0.82-1.28)  All: 0.87 (0.73-1.04)  M: 0.80 (0.92-1.04) F: 0.96 (0.76-1.21) | 0.62  0.90  0.66  0.012  0.036  0.007  0.08  0.028  0.86  0.35  0.25  0.83  0.13  0.10  0.71 |
| Chen et al.^16^  (Included Mozaffarian et al. and Bell et al. reports in the table) | 2016 | Meta-analysis/ general populations | All-cause mortality | 11 studies  371,965  Dietary intakes (n=7)  361,273  Circulating levels (n=4)  10,692 | 31,185  27,624  3,561 | Circulating EPA  Circulating DHA | Summary RR (Highest to lowest; 95% CI) | 0.74 (0.60-0.90)  0.78 (0.64-0.93) | NR |
| Delgado et al.^17^ | 2017 | Prospective cohort/ patients referred for coronary angiography, LURIC study, German | All-cause mortality  CVD mortality | 3259 | 975  614 | Plasma total omega-6 PUFA  GLA  ADA  DPA | 1 SD increase (95 % CI) | All: 0.93 (0.87-0.99)  CVD: 0.95 (0.87-1.03)  All: 0.88 (0.82-0.95)  CVD: 0.86 (0.79-0.95)  All: 1.10 (1.03-1.18)  CVD: 1.12 (1.04-1.22)  All: 1.12 (1.05-1.19)  CVD: 1.11 (1.02-1.20) | NR |
| Hamazaki et al.^18^ | 2018 | Nested case-control study/ JPHC study, Japan | Coronary heart disease | 209 cases  418 matched controls | (168 myocardial infarction  41 sudden cardiac death)  (157 non-fatal coronary events  52 fatal) | Plasma n-3 PUFAs | OR in quartiles (Highest to lowest; 95% CI) | CHD:0.79 (0.41-1.51)  MI: 0.91 (0.43-1.89)  Sudden death: 0.08 (0.01-0.88)  Non-fatal: 0.89 (0.42-1.89) Fatal: 0.12 (0.02-0.75) | 0.51  0.90  0.04  0.97  0.03 |
| Harris et al.^19^ | 2018 | Prospective cohort study/ the Framingham Heart Study Offspring cohort, US | All-cause mortality  CVD mortality  Cancer mortality  CVD events | 2500 | 350  58  146  245 | Omega-3 index  red blood cells | HR in quintiles (Highest to lowest; 95% CI) | All-death: 0.66 (0.45-0.96)  CVD mortality: 0.39 (0.15-1.02)  Cancer mortality: 0.96 (0.56-1.64)  CVD events: 0.61 (0.37-0.99) | 0.02  0.10  0.88  0.008 |
| Kamleh et al.^20^ | 2018 | Case-control study/ IMPROVE pan-European cohort, age 55-79 | Incidence of CVD events | Cases: 173  Matched controls: 172  Diabetics  Cases: 40  Matched controls: 39  Non-diabetics  Cases: 133  Matched controls: 133 | - | Plasma  DHA  GLA  AA  ADA | HR per 1-SD increase in log transformed metabolite concentration (95% CI) | Diabetics: NS  Non-diabetics:  DHA: 0.86 (0.72-1.02)  GLA: 0.72 (0.59-0.88)  AA: 0.83 (0.70-0.99)  ADA: 0.79 (0.66-0.95) | 0.085  0.001  0.037  0.012 |
| Miura et al.^21^ | 2018 | Community-based prospective cohort/ women, 25-75 age, Australia | All-cause mortality | 564 | 81 | Plasma  Omega-3  Omega-6 | HR in tertiles (Highest to lowest; 95% CI) | Absolute (ug/ml)  Omega-3: 1.05 (0.57-1.92)  Omega-6: 1.19 (0.65-2.18)  Relative (%)  Omega-3: 0.97 (0.54-1.72)  Omega-6: 1.02 (0.58-1.78) | 0.84  0.60  0.77  1.00 |
| Marklund et al.^22^ | 2019 | Meta-analysis | Total CVD  CVD mortality  Total CHD  Ischemic stroke | 30 prospective studies:  18 cohort  12 nested case-control or  case-control | 10,477  4,508  11,857  3,705 | Circulating  LA  AA | HR per interquintile range (95% CI) | LA  Total CVD: 0.93 (0.88-0.99)  CVD mortality: 0.78 (0.70-0.85)  Total CHD: 0.94 (0.88-1.00)  Ischemic stroke: 0.88 (0.79-0.98)  AA  Total CVD: 0.95 (0.90-1.01)  CVD mortality: 0.94 (0.86-1.02)  Total CHD: 0.99 (0.94-1.04)  Ischemic stroke: 0.99 (0.90-1.10) | NR |
| Harris et al.^23^ | 2020 | Case-cohort study/ secondary analysis of ADVANCE study, patients with type 2 diabetes | Macrovascular disease  Microvascular disease  All-cause mortality  CVD mortality | 3,576 | 654  341  631  330 | Plasma  Omega-3  Omega-6 | HR per 1-SD increase of the percentage contribution of total fatty acids (95% CI) | Macrovascular disease  Omega-3: 0.87 (0.80-0.95)  Omega-6: 0.97 (0.89-1.07)  Microvascular disease  Omega-3: 1.01 (0.91-1.13)  Omega-6: 0.97 (0.86-1.10)  All-cause mortality  Omega-3: 0.91 (0.84-0.99)  Omega-6: 0.97 (0.88-1.07)  CVD mortality  Omega-3: 0.85 (0.75-0.96) | NR |
| Harris et al.^24^ | 2021 | Meta-analysis/ age 50-81 | All-cause mortality  CVD mortality  Cancer mortality | All: 17 cohorts, 42,466  CVD: 15 cohorts  Cancer: 15 cohorts | 15,720  4,571  4,284 | Serum  EPA  DPA  DHA  EPA+DHA | HR in quintiles (Highest to lowest; 95% CI) | EPA  All: 0.82 (0.78-0.87)  CVD: 0.85 (0.77-0.94)  Cancer: 0.82 (0.74-0.91)  DPA  All: 0.84 (0.79-0.90)  CVD: 0.87 (0.78-0.98)  Cancer: 0.79 (0.70-0.90)  DHA  All: 0.85 (0.81-0.90)  CVD: 0.79 (0.72-0.88)  Cancer: 0.86 (0.78-0.95)  EPA+DHA  All: 0.84 (0.79-0.89)  CVD: 0.80 (0.73-0.88)  Cancer: 0.87 (0.78-0.96) | <0.001  0.006  0.008  <0.001  0.16  0.008  0.01  0.002  0.06  <0.001  <0.001  0.06 |
| Kamalita et al.^25^ | 2021 | Prospective cohort/ patients with prior MI, 60-80 years old, Dutch | All-cause mortality  CVD mortality | 4,067 | 1,877  834 | Circulating EPA + DHA | HR in quintiles (Highest to lowest; 95% CI) | 0.73 (0.63-0.86) 0.75 (0.60-0.95) | <0.001  0.016 |
| Diffenderfer et al.^26^ | 2022 | Plasma fatty acid profiles in US | Heart disease mortality rate | 1,169,621 | NR | Plasma EPA  Omega-3 index | Pearson correlation coefficient | -0.504  -0.570 | <0.001  <0.001 |
| *Dietary PUFAs* | | | | | | | | | |
| Bell et al.^27^ | 2014 | Population based prospective cohort/ VITAL Study, 50-76 yrs old, western Washington State, recruited 2000-2002 | All-cause mortality  CVD mortality  Cancer mortality | 70,495 | 3,051  769  1,485 | EPA + DHA intake (diet + supplements) | HR in quartiles (Highest to lowest; 95% CI) | 0.82 (0.73-0.93)  0.87 (0.68-1.10)  0.77 (0.64-0.92) | 0.001  0.158  0.001 |
| Chen et al.^16^  (Included Mozaffarian et al. and Bell et al. reports in the table) | 2016 | Meta-analysis/ general populations | All-cause mortality | 11 studies  371,965  Dietary intakes (n=7)  361,273  Circulating levels (n=4)  10,692 | 31,185  27,624  3,561 | N-3 LCPUFA intake | Summary RR (Highest to lowest; 95% CI) | 0.91 (0.84-0.98) | NR |
| Zhang et al.^28^ | 2018 | Population based prospective cohort/ NIH-AARP Diet and Health Study, US | All-cause mortality  CVD mortality  Cancer mortality | 421,309  M: 240,729  F: 180,580 | All  M: 54,230  F: 30,882  CVD  M: 14,824 F: 7,541  Cancer  M: 20,041 F: 11,526 | Long-chain omega-3 PUFA intake | HR in quintiles (Highest to lowest; 95% CI) | All  M: 0.89 (0.86-0.92)  F: 0.90 (0.86-0.94)  CVD:  M: 0.85 (0.80-0.90)  F: 0.82 (0.75-0.90) Cancer: M: 0.95 (0.90-1.00) F: 1.01 (0.93-1.09) | < 0.001  < 0.001  <0.001  < 0.001  0.04  0.51 |
| Kamalita et al.^25^ | 2021 | Prospective cohort/ patients with prior MI, 60-80 years old, Dutch | All-cause mortality  CVD mortality | 4,067 | 1,877  834 | Dietary EPA + DHA intake | HR in quintiles (Highest to lowest; 95% CI) | All: 0.86 (0.75-0.99)  CVD: 0.84 (0.68-1.04) | 0.11  0.22 |
| *Fish intake and fish oil supplementation* | | | | | | | | | |
| Nagata et al.^29^ | 2002 | Population based prospective cohort/ Japan, age >= 35, 1992-1999 | All-cause mortality  CVD mortality  Cancer mortality | 29,079  M: 13,355  F: 15,724 | 2,062  M: 1,163  F: 899  635  M: 308  F: 327  653  M: 400  F: 253 | Fish oil | HR in quintiles (Highest to lowest; 95% CI) | M: 0.87 (0.73-1.05)  F: 0.77 (0.62-0.94)  M: 0.76 (0.54-1.07)  F: 0.77 (0.55-1.00)  M: 0.89 (0.66-1.20)  F: 0.70 (0.47-1.05) | 0.38  0.01  0.27  0.16  0.52  0.15 |
| Li et al.^30^ | 2020 | Population based prospective cohort/ UK biobank | All-cause mortality  CVD mortality | 427,678 | 12,928  3282 | Fish oil supplementation | HR (Yes to No; 95% CI) | 0.87 (0.83-0.90)  0.84 (0.78-0.91) | NR |
| Jayedi et al.^31^ | 2021 | Meta-analysis/ patients with type 2 diabetes | All-cause mortality  CVD diseases  CHD  MI  stroke | Total:  9 studies (57,394)  8 studies (57,077)  4 studies (8781) | NR  791  376  372 | Fish consumption | Pooled RR (Highest to lowest; 95% CI) | 0.86 (0.76-0.96)  0.61 (0.29-0.93)  NS  NS | NR |
| Liu et al.^32^ | 2022 | Population based prospective cohort/ UK Biobank, UK | Overall cancer | 470,804 | 28,417 | Fish oil supplementation | HR (Yes to No; 95% CI) | 0.97 (0.95-1.00) | 0.06 |
| Ma et al.^33^ | 2022 | Prospective cohort/ patients with hypertension, UK Biobank, UK | Incidence of cardiometabolic multimorbidity (CMM)  All-cause mortality  CVD mortality  Cancer mortality | 81,579 | 15,990  6,456  1,308  3,307 | Fish oil supplementation | HR (Yes to No; 95% CI) | 0.92 (0.89-0.96)  0.90 (0.85-0.95)  0.86 (0.76-0.98)  0.99 (0.91-1.07) | <0.001  <0.001  0.027  0.742 |
| Abbreviations: HR, hazards ratio; NR, not recorded; NS, not significant. | | | | | | | | | |

**References**

1. Noori N, Dukkipati R, Kovesdy CP, et al. Dietary omega-3 fatty acid, ratio of omega-6 to omega-3 intake, inflammation, and survival in long-term hemodialysis patients. *Am J of Kidney Dis.* 2011;58(2):248-256.

2. Harris WS, Luo J, Pottala JV, et al. Red blood cell polyunsaturated fatty acids and mortality in the Women's Health Initiative Memory Study. *J Clin Lipidol.* 2017;11(1):250-259.e255.

3. Otsuka R, Tange C, Nishita Y, et al. Fish and meat intake, serum eicosapentaenoic acid and docosahexaenoic acid levels, and mortality in community-dwelling Japanese older persons. *Int J Environ Res Public Health.* 2019;16(10).

4. Zhuang P, Wang W, Wang J, Zhang Y, Jiao J. Polyunsaturated fatty acids intake, omega-6/omega-3 ratio and mortality: Findings from two independent nationwide cohorts. *Clin Nutr.* 2019;38(2):848-855.

5. Wang Y, Crawford MA, Chen J, et al. Fish consumption, blood docosahexaenoic acid and chronic diseases in Chinese rural populations. *Comp Biochem Physiol A Mol Integr Physiol.* 2003;136(1):127-140.

6. Lindberg M, Saltvedt I, Sletvold O, Bjerve KS. Long-chain n-3 fatty acids and mortality in elderly patients. *Am J Clin Nutr.* 2008;88(3):722-729.

7. Chattipakorn N, Settakorn J, Petsophonsakul P, et al. Cardiac mortality is associated with low levels of omega-3 and omega-6 fatty acids in the heart of cadavers with a history of coronary heart disease. *Nutr Res.* 2009;29(10):696-704.

8. Lee S-H, Shin M-J, Kim J-S, et al. Blood eicosapentaenoic acid and docosahexaenoic acid as predictors of all-cause mortality in patients with acute myocardial infarction-data from Infarction Prognosis Study (IPS) Registry. *Circ J.* 2009;73(12):2250-2257.

9. Pottala JV, Garg S, Cohen BE, Whooley MA, Harris WS. Blood eicosapentaenoic and docosahexaenoic acids predict all-cause mortality in patients with stable coronary heart disease: the Heart and Soul study. *Circ Cardiovasc Qual Outcomes.* 2010;3(4):406-412.

10. Petrone AB, Weir N, Hanson NQ, et al. Omega-6 fatty acids and risk of heart failure in the Physicians’ Health Study. *Am J Clin Nutr.* 2012;97(1):66-71.

11. Mozaffarian D, Lemaitre RN, King IB, et al. Plasma phospholipid long-chain ω-3 fatty acids and total and cause-specific mortality in older adults: a cohort study. *Ann Intern Med.* 2013;158(7):515-525.

12. Wu JHY, Lemaitre RN, King IB, et al. Circulating omega-6 polyunsaturated fatty acids and total and cause-specific mortality. *Circulation.* 2014;130(15):1245-1253.

13. Eide IA, Dahle DO, Svensson M, et al. Plasma levels of marine n-3 fatty acids and cardiovascular risk markers in renal transplant recipients. *Eur J Clin Nutr.* 2016;70(7):824-830.

14. Kleber ME, Delgado GE, Lorkowski S, März W, von Schacky C. Omega-3 fatty acids and mortality in patients referred for coronary angiography. The Ludwigshafen Risk and Cardiovascular Health Study. *Atherosclerosis.* 2016;252:175-181.

15. Miura K, Hughes MCB, Ungerer JPJ, Green AC. Plasma eicosapentaenoic acid is negatively associated with all-cause mortality among men and women in a population-based prospective study. *Nutr Res.* 2016;36(11):1202-1209.

16. Chen GC, Yang J, Eggersdorfer M, Zhang W, Qin LQ. N-3 long-chain polyunsaturated fatty acids and risk of all-cause mortality among general populations: a meta-analysis. *Sci Rep.* 2016;6:28165.

17. Delgado GE, März W, Lorkowski S, von Schacky C, Kleber ME. Omega-6 fatty acids: opposing associations with risk—The Ludwigshafen Risk and Cardiovascular Health Study. *J Clin Lipidol.* 2017;11(4):1082-1090.e1014.

18. Hamazaki K, Iso H, Eshak ES, et al. Plasma levels of n-3 fatty acids and risk of coronary heart disease among Japanese: The Japan Public Health Center-based (JPHC) study. *Atherosclerosis.* 2018;272:226-232.

19. Harris WS, Tintle NL, Etherton MR, Vasan RS. Erythrocyte long-chain omega-3 fatty acid levels are inversely associated with mortality and with incident cardiovascular disease: The Framingham Heart Study. *J Clin Lipidol.* 2018;12(3):718-727.e716.

20. Kamleh MA, McLeod O, Checa A, et al. Increased levels of circulating fatty acids are associated with protective effects against future cardiovascular events in nondiabetics. *J Proteome Res.* 2018;17(2):870-878.

21. Miura K, Hughes MCB, Ungerer JPJ, Smith DD, Green AC. Absolute versus relative measures of plasma fatty acids and health outcomes: example of phospholipid omega-3 and omega-6 fatty acids and all-cause mortality in women. *Eur J Nutr.* 2018;57(2):713-722.

22. Marklund M, Wu JHY, Imamura F, et al. Biomarkers of dietary omega-6 fatty acids and incident cardiovascular disease and mortality. *Circulation.* 2019;139(21):2422-2436.

23. Harris K, Oshima M, Sattar N, et al. Plasma fatty acids and the risk of vascular disease and mortality outcomes in individuals with type 2 diabetes: results from the ADVANCE study. *Diabetologia.* 2020;63(8):1637-1647.

24. Harris WS, Tintle NL, Imamura F, et al. Blood n-3 fatty acid levels and total and cause-specific mortality from 17 prospective studies. *Nat Commun.* 2021;12(1):2329.

25. Pertiwi K, Küpers LK, Goede Jd, Zock PL, Kromhout D, Geleijnse JM. Dietary and circulating long-chain omega-3 polyunsaturated fatty acids and mortality risk after myocardial infarction: a long-term follow-up of the Alpha Omega Cohort. *J Am Heart Assoc.* 2021;10(23):e022617.

26. Diffenderfer MR, Rajapakse N, Pham E, et al. Plasma fatty acid profiles: relationships with sex, age, and state-reported heart disease mortality rates in the United States. *J Clin Lipidol.* 2022;16(2):184-197.

27. Bell GA, Kantor ED, Lampe JW, Kristal AR, Heckbert SR, White E. Intake of long-chain ω-3 fatty acids from diet and supplements in relation to mortality. *Am J Epidemiol.* 2014;179(6):710-720.

28. Zhang Y, Zhuang P, He W, et al. Association of fish and long-chain omega-3 fatty acids intakes with total and cause-specific mortality: prospective analysis of 421 309 individuals. *J Intern Med.* 2018;284(4):399-417.

29. Nagata C, Takatsuka N, Shimizu H. Soy and fish oil intake and mortality in a Japanese community. *Am J Epidemiol.* 2002;156(9):824-831.

30. Li Z-H, Zhong W-F, Liu S, et al. Associations of habitual fish oil supplementation with cardiovascular outcomes and all cause mortality: evidence from a large population based cohort study. *BMJ.* 2020;368:m456.

31. Jayedi A, Soltani S, Abdolshahi A, Shab-Bidar S. Fish consumption and the risk of cardiovascular disease and mortality in patients with type 2 diabetes: a dose-response meta-analysis of prospective cohort studies. *Crit Rev Food Sci Nutr.* 2021;61(10):1640-1650.

32. Liu Z, Luo Y, Ren J, et al. Association between fish oil supplementation and cancer risk according to fatty fish consumption: A large prospective population-based cohort study using UK Biobank. *Int J Cancer.* 2022;150(4):562-571.

33. Ma T, He L, Luo Y, et al. Associations of baseline use of fish oil with progression of cardiometabolic multimorbidity and mortality among patients with hypertension: a prospective study of UK Biobank. *Eur J Nutr.* 2022.
