# Supplementary material for: Higher ratio of plasma omega-6/omega-3 fatty acids is associated with greater risk of all-cause, cancer, and cardiovascular mortality: A population-based cohort study in UK Biobank
Source: eLife. 2024 Apr 5;12:RP90132. doi: 10.7554/eLife.90132 (PMC10997328; doi:10.7554/eLife.90132)
Supplement: Supplementary file 2. [file elife-90132-supp2.docx]

**Table S2.** Risk estimates^a^ of ratio of plasma omega-6 to omega-3 PUFAs with all-cause, cancer and CVD mortality, stratified by potential risk factors, in the UK Biobank Study (n = 85,425).

| **Stratification variables and ratio quintiles** | **Causes of death** | | | | | | | |
| --- | --- | --- | --- | --- | --- | --- | --- | --- |
|  | **All-cause** | |  | **Cancer** | |  | **Cardiovascular diseases** | |
|  | Death (n) | HR (95% CI) |  | Death (n) | HR (95% CI) |  | Death (n) | HR (95% CI) |
| **Age, years** |  |  |  |  |  |  |  |  |
| **Continuous (p for interaction)** | 0.224 | |  | 0.118 | |  | 0.875 | |
| **< 58** |  |  |  |  |  |  |  |  |
| 1 | 194 | 1.00 (ref) |  | 85 | 1.00 (ref) |  | 56 | 1.00 (ref) |
| 2 | 223 | 0.92 (0.74-1.14) |  | 118 | 1.08 (0.79-1.47) |  | 47 | 0.74 (0.47-1.14) |
| 3 | 284 | 0.92 (0.75-1.13) |  | 129 | 0.92 (0.68-1.26) |  | 79 | 0.98 (0.66-1.45) |
| 4 | 305 | 0.93 (0.76-1.14) |  | 154 | 1.05 (0.78-1.41) |  | 71 | 0.80 (0.53-1.20) |
| 5 | 430 | 1.11 (0.91-1.34) |  | 188 | 1.07 (0.80-1.43) |  | 112 | 1.11 (0.77-1.61) |
| *P for trend* |  | *0.070* |  |  | *0.611* |  |  | *0.193* |
| **≥ 58** |  |  |  |  |  |  |  |  |
| 1 | 1154 | 1.00 (ref) |  | 508 | 1.00 (ref) |  | 313 | 1.00 (ref) |
| 2 | 1033 | 0.95 (0.86-1.04) |  | 445 | 0.94 (0.81-1.08) |  | 268 | 0.92 (0.76-1.11) |
| 3 | 952 | 0.98 (0.89-1.09) |  | 414 | 0.96 (0.83-1.11) |  | 242 | 0.92 (0.75-1.12) |
| 4 | 947 | 1.06 (0.96-1.17) |  | 394 | 1.06 (0.91-1.23) |  | 235 | 1.03 (0.84-1.25) |
| 5 | 939 | 1.17 (1.06-1.29) |  | 359 | 1.04 (0.89-1.21) |  | 245 | 1.24 (1.02-1.51) |
| *P for trend* |  | *< 0.001* |  |  | *0.317* |  |  | *0.010* |
| *P for interaction* |  | *0.798* |  |  | *0.853* |  |  | *0.588* |
|  |  |  |  |  |  |  |  |  |
| **Sex** |  |  |  |  |  |  |  |  |
| **Male** |  |  |  |  |  |  |  |  |
| 1 | 730 | 1.00 (ref) |  | 287 | 1.00 (ref) |  | 236 | 1.00 (ref) |
| 2 | 755 | 0.99 (0.89-1.11) |  | 317 | 1.08 (0.91-1.29) |  | 217 | 0.88 (0.72-1.09) |
| 3 | 777 | 1.01 (0.90-1.13) |  | 301 | 0.99 (0.83-1.19) |  | 227 | 0.91 (0.73-1.12) |
| 4 | 834 | 1.11 (0.99-1.24) |  | 338 | 1.15 (0.96-1.37) |  | 224 | 1.02 (0.83-1.25) |
| 5 | 980 | 1.29 (1.16-1.44) |  | 355 | 1.17 (0.98-1.40) |  | 282 | 1.31 (1.08-1.60) |
| *P for trend* |  | *< 0.001* |  |  | *0.057* |  |  | *< 0.001* |
| **Female** |  |  |  |  |  |  |  |  |
| 1 | 618 | 1.00 (ref) |  | 306 | 1.00 (ref) |  | 133 | 1.00 (ref) |
| 2 | 501 | 0.91 (0.79-1.05) |  | 246 | 0.85 (0.70-1.04) |  | 98 | 0.92 (0.66-1.27) |
| 3 | 459 | 1.03 (0.89-1.19) |  | 242 | 1.00 (0.81-1.22) |  | 94 | 1.18 (0.85-1.64) |
| 4 | 418 | 1.07 (0.92-1.25) |  | 210 | 1.08 (0.88-1.33) |  | 82 | 1.07 (0.76-1.52) |
| 5 | 389 | 1.22 (1.04-1.42) |  | 192 | 1.11 (0.89-1.38) |  | 75 | 1.31 (0.91-1.88) |
| *P for trend* |  | *0.003* |  |  | *0.110* |  |  | *0.099* |
| *P for interaction* |  | *0.798* |  |  | *0.333* |  |  | *0.731* |
|  |  |  |  |  |  |  |  |  |
| **TDI** |  |  |  |  |  |  |  |  |
| **Continuous (p for interaction)** | 0.196 | |  | 0.351 | |  | 0.945 | |
| **< -2** |  |  |  |  |  |  |  |  |
| 1 | 719 | 1.00 (ref) |  | 352 | 1.00 (ref) |  | 180 | 1.00 (ref) |
| 2 | 582 | 0.94 (0.83-1.06) |  | 282 | 0.88 (0.74-1.05) |  | 129 | 0.85 (0.66-1.11) |
| 3 | 574 | 1.00 (0.88-1.13) |  | 281 | 0.92 (0.77-1.10) |  | 139 | 1.03 (0.80-1.34) |
| 4 | 549 | 1.07 (0.95-1.22) |  | 268 | 1.04 (0.87-1.24) |  | 128 | 1.11 (0.85-1.44) |
| 5 | 500 | 1.17 (1.02-1.33) |  | 240 | 1.06 (0.88-1.28) |  | 121 | 1.35 (1.04-1.75) |
| *P for trend* |  | *0.004* |  |  | *0.240* |  |  | *0.005* |
| **≥ -2** |  |  |  |  |  |  |  |  |
| 1 | 629 | 1.00 (ref) |  | 241 | 1.00 (ref) |  | 189 | 1.00 (ref) |
| 2 | 673 | 0.99 (0.87-1.12) |  | 280 | 1.14 (0.93-1.39) |  | 186 | 0.93 (0.73-1.18) |
| 3 | 662 | 1.05 (0.93-1.20) |  | 262 | 1.11 (0.91-1.36) |  | 182 | 0.95 (0.74-1.21) |
| 4 | 702 | 1.13 (1.00-1.28) |  | 280 | 1.25 (1.02-1.53) |  | 178 | 0.98 (0.77-1.25) |
| 5 | 868 | 1.38 (1.22-1.55) |  | 307 | 1.29 (1.06-1.58) |  | 235 | 1.32 (1.05-1.66) |
| *P for trend* |  | *< 0.001* |  |  | 0.010 |  |  | *0.004* |
| *P for interaction* |  | *0.268* |  |  | *0.395* |  |  | *0.846* |
| **BMI** | 0.925 | |  | 0.822 | |  | 0.298 | |
| **Continuous (p for interaction)** |  |  |  |  |  |  |  |  |
| **< 25** |  |  |  |  |  |  |  |  |
| 1 | 373 | 1.00 (ref) |  | 179 | 1.00 (ref) |  | 79 | 1.00 (ref) |
| 2 | 298 | 0.90 (0.76-1.07) |  | 139 | 0.91 (0.71-1.16) |  | 66 | 0.92 (0.63-1.33) |
| 3 | 305 | 0.93 (0.78-1.11) |  | 144 | 0.85 (0.66-1.10) |  | 59 | 0.89 (0.61-1.31) |
| 4 | 333 | 0.98 (0.83-1.17) |  | 151 | 1.05 (0.82-1.34) |  | 66 | 0.87 (0.59-1.27) |
| 5 | 429 | 1.15 (0.97-1.36) |  | 184 | 1.04 (0.81-1.34) |  | 97 | 1.32 (0.93-1.88) |
| *P for trend* |  | *0.025* |  |  | *0.399* |  |  | *0.071* |
| **≥ 25** |  |  |  |  |  |  |  |  |
| 1 | 965 | 1.00 (ref) |  | 413 | 1.00 (ref) |  | 286 | 1.00 (ref) |
| 2 | 948 | 0.99 (0.89-1.10) |  | 422 | 1.02 (0.87-1.18) |  | 244 | 0.90 (0.73-1.10) |
| 3 | 917 | 1.05 (0.95-1.17) |  | 396 | 1.06 (0.91-1.24) |  | 261 | 1.02 (0.83-1.24) |
| 4 | 907 | 1.14 (1.03-1.26) |  | 392 | 1.15 (0.98-1.35) |  | 236 | 1.09 (0.89-1.33) |
| 5 | 927 | 1.30 (1.17-1.44) |  | 362 | 1.19 (1.01-1.40) |  | 257 | 1.30 (1.07-1.59) |
| *P for trend* |  | *< 0.001* |  |  | 0.012 |  |  | 0.001 |
| *P for interaction* |  | *0.598* |  |  | *0.782* |  |  | *0.615* |
|  |  |  |  |  |  |  |  |  |
| **Comorbidities** |  |  |  |  |  |  |  |  |
| **Yes** |  |  |  |  |  |  |  |  |
| 1 | 878 | 1.00 (ref) |  | 329 | 1.00 (ref) |  | 285 | 1.00 (ref) |
| 2 | 806 | 0.95 (0.85-1.06) |  | 302 | 0.99 (0.83-1.18) |  | 239 | 0.86 (0.70-1.05) |
| 3 | 740 | 0.95 (0.85-1.06) |  | 270 | 0.95 (0.79-1.14) |  | 220 | 0.84 (0.67-1.03) |
| 4 | 797 | 1.09 (0.98-1.22) |  | 276 | 1.05 (0.87-1.26) |  | 242 | 1.09 (0.89-1.33) |
| 5 | 849 | 1.27 (1.14-1.42) |  | 282 | 1.12 (0.93-1.35) |  | 250 | 1.24 (1.01-1.52) |
| *P for trend* |  | *< 0.001* |  |  | 0.172 |  |  | 0.002 |
| **No** |  |  |  |  |  |  |  |  |
| 1 | 470 | 1.00 (ref) |  | 264 | 1.00 (ref) |  | 84 | 1.00 (ref) |
| 2 | 450 | 0.97 (0.84-1.12) |  | 261 | 0.98 (0.80-1.18) |  | 76 | 0.99 (0.70-1.42) |
| 3 | 496 | 1.12 (0.97-1.29) |  | 273 | 1.05 (0.86-1.27) |  | 101 | 1.37 (0.98-1.91) |
| 4 | 455 | 1.09 (0.94-1.26) |  | 272 | 1.20 (0.99-1.45) |  | 64 | 0.82 (0.56-1.20) |
| 5 | 520 | 1.24 (1.07-1.43) |  | 265 | 1.17 (0.96-1.43) |  | 107 | 1.47 (1.05-2.06) |
| *P for trend* |  | *0.001* |  |  | *0.027* |  |  | *0.046* |
| *P for interaction* |  | *0.219* |  |  | *0.736* |  |  | *0.006* |
|  |  |  |  |  |  |  |  |  |
| **Physical activity** |  |  |  |  |  |  |  |  |
| **Low or moderate** |  |  |  |  |  |  |  |  |
| 1 | 686 | 1.00 (ref) |  | 303 | 1.00 (ref) |  | 175 | 1.00 (ref) |
| 2 | 626 | 0.96 (0.86-1.07) |  | 271 | 0.97 (0.82-1.14) |  | 158 | 0.93 (0.74-1.15) |
| 3 | 582 | 1.01 (0.90-1.13) |  | 251 | 1.01 (0.85-1.19) |  | 136 | 0.89 (0.71-1.12) |
| 4 | 617 | 1.11 (1.00-1.24) |  | 267 | 1.12 (0.95-1.33) |  | 162 | 1.10 (0.88-1.36) |
| 5 | 651 | 1.30 (1.17-1.46) |  | 247 | 1.17 (0.98-1.39) |  | 180 | 1.37 (1.10-1.70) |
| *P for trend* |  | *< 0.001* |  |  | 0.020 |  |  | *< 0.001* |
| **High** |  |  |  |  |  |  |  |  |
| 1 | 388 | 1.00 (ref) |  | 183 | 1.00 (ref) |  | 100 | 1.00 (ref) |
| 2 | 346 | 0.95 (0.82-1.10) |  | 172 | 1.01 (0.82-1.24) |  | 77 | 0.82 (0.61-1.11) |
| 3 | 352 | 1.04 (0.90-1.21) |  | 157 | 0.98 (0.79-1.22) |  | 99 | 1.15 (0.87-1.53) |
| 4 | 341 | 1.06 (0.91-1.23) |  | 168 | 1.10 (0.89-1.36) |  | 76 | 0.92 (0.68-1.25) |
| 5 | 383 | 1.20 (1.04-1.39) |  | 165 | 1.11 (0.89-1.38) |  | 100 | 1.22 (0.91-1.62) |
| *P for trend* |  | 0.003 |  |  | *0.228* |  |  | *0.099* |
| *P for interaction* |  | *0.883* |  |  | *0.994* |  |  | *0.188* |
| **Smoke status** |  |  |  |  |  |  |  |  |
| **Yes** |  |  |  |  |  |  |  |  |
| 1 | 155 | 1.00 (ref) |  | 65 | 1.00 (ref) |  | 41 | 1.00 (ref) |
| 2 | 193 | 1.01 (0.79-1.30) |  | 82 | 0.95 (0.65-1.37) |  | 56 | 1.47 (0.90-2.38) |
| 3 | 249 | 1.22 (0.96-1.54) |  | 96 | 0.98 (0.68-1.42) |  | 71 | 1.53 (0.95-2.47) |
| 4 | 292 | 1.24 (0.98-1.56) |  | 132 | 1.23 (0.87-1.74) |  | 74 | 1.55 (0.97-2.49) |
| 5 | 438 | 1.57 (1.26-1.95) |  | 176 | 1.41 (1.01-1.97) |  | 116 | 2.09 (1.33-3.27) |
| *P for trend* |  | *< 0.001* |  |  | *0.003* |  |  | *0.001* |
| **No** |  |  |  |  |  |  |  |  |
| 1 | 1178 | 1.00 (ref) |  | 523 | 1.00 (ref) |  | 322 | 1.00 (ref) |
| 2 | 1054 | 0.96 (0.87-1.05) |  | 479 | 0.99 (0.86-1.14) |  | 256 | 0.83 (0.69-1.00) |
| 3 | 973 | 0.98 (0.89-1.08) |  | 441 | 1.00 (0.86-1.15) |  | 247 | 0.90 (0.74-1.09) |
| 4 | 949 | 1.06 (0.97-1.17) |  | 413 | 1.09 (0.94-1.26) |  | 231 | 0.96 (0.79-1.17) |
| 5 | 918 | 1.18 (1.06-1.30) |  | 367 | 1.06 (0.91-1.23) |  | 238 | 1.18 (0.97-1.43) |
| *P for trend* |  | *< 0.001* |  |  | *0.264* |  |  | *0.024* |
| *P for interaction* |  | *0.007* |  |  | *0.125* |  |  | *0.116* |
|  |  |  |  |  |  |  |  |  |

Abbreviations: CI, confidence interval; HR, hazards ratio; ref, reference.

^a^ From Cox proportional hazards regression; adjusted for age (years; continuous), sex (male, female), race (White, Black, Asian, Others), Townsend deprivation index (continuous), assessment centre, BMI (kg/m2; continuous), smoking status (never, previous, current), alcohol intake status (never, previous, current), physical activity (low, moderate, high), and comorbidities (yes, no).

**Table S3.** Associations^a^ of plasma omega-3 PUFAs percentage with all-cause, cancer, and CVD mortality risk in the UK Biobank Study.

| **Omega ratio variable forms** | **Causes of death** | | | | | | | | | | | | | |
| --- | --- | --- | --- | --- | --- | --- | --- | --- | --- | --- | --- | --- | --- | --- |
|  | **All-cause** | | | |  | **Cancer** | | | |  | **Cardiovascular diseases** | | | |
|  | Death # | Model 1^b^ | Model 2^c^ | Model 3^d^ |  | Death # | Model 1^b^ | Model 2^c^ | Model 3^d^ |  | Death # | Model 1^b^ | Model 2^c^ | Model 3^d^ |
|  |  | HR  (95% CI) | HR  (95% CI) | HR  (95% CI) |  |  | HR  (95% CI) | HR  (95% CI) | HR  (95% CI) |  |  | HR  (95% CI) | HR  (95% CI) | HR  (95% CI) |
| Continuous | 6,461 | 0.91 | 0.90 | 0.94 |  | 2,794 | 0.93 | 0.92 | 0.95 |  | 1,668 | 0.92 | 0.90 | 0.93 |
|  |  | (0.89-0.92) | (0.88-0.91) | (0.92-0.95) |  |  | (0.90-0.95) | (0.89-0.94) | (0.93-0.98) |  |  | (0.89-0.95) | (0.87-0.94) | (0.90-0.97) |
| Quintiles (median) | |  |  |  |  |  |  |  |  |  |  |  |  |  |
| 1 (2.7) | 1,526 | 1.00 | 1.00 | 1.00 |  | 600 | 1.00 | 1.00 | 1.00 |  | 413 | 1.00 | 1.00 | 1.00 |
|  |  | (ref) | (ref) | (ref) |  |  | (ref) | (ref) | (ref) |  |  | (ref) | (ref) | (ref) |
| 2 (3.5) | 1,283 | 0.81 | 0.78 | 0.82 |  | 542 | 0.85 | 0.83 | 0.89 |  | 327 | 0.77 | 0.74 | 0.75 |
|  |  | (0.75-0.87) | (0.72-0.84) | (0.76-0.90) |  |  | (0.76-0.96) | (0.74-0.94) | (0.78-1.01) |  |  | (0.67-0.90) | (0.64-0.86) | (0.64-0.89) |
| 3 (4.2) | 1,246 | 0.75 | 0.72 | 0.77 |  | 581 | 0.86 | 0.84 | 0.91 |  | 300 | 0.69 | 0.65 | 0.67 |
|  |  | (0.70-0.81) | (0.67-0.78) | (0.71-0.84) |  |  | (0.77-0.97) | (0.75-0.94) | (0.79-1.04) |  |  | (0.59-0.80) | (0.56-0.75) | (0.56-0.80) |
| 4 (4.9) | 1,244 | 0.71 | 0.68 | 0.76 |  | 553 | 0.77 | 0.75 | 0.84 |  | 323 | 0.73 | 0.68 | 0.72 |
|  |  | (0.66-0.77) | (0.63-0.73) | (0.69-0.83) |  |  | (0.69-0.87) | (0.66-0.84) | (0.73-0.96) |  |  | (0.63-0.85) | (0.59-0.79) | (0.60-0.85) |
| 5 (6.3) | 1,162 | 0.62 | 0.59 | 0.69 |  | 518 | 0.67 | 0.64 | 0.75 |  | 305 | 0.65 | 0.61 | 0.68 |
|  |  | (0.57-0.67) | (0.54-0.64) | (0.63-0.76) |  |  | (0.59-0.76) | (0.57-0.73) | (0.65-0.87) |  |  | (0.56-0.76) | (0.52-0.71) | (0.57-0.82) |
| *P*_trend_ |  | *<0.001* | *<0.001* | *<0.001* |  |  | *<0.001* | *<0.001* | *<0.001* |  |  | *<0.001* | *<0.001* | *<0.001* |

Abbreviations: CI, confidence interval; HR, hazards ratio; ref, reference.

^a^ From Cox proportional hazards regression.

^b^ Adjusted for age (years; continuous), sex (male, female), race (White, Black, Asian, Others), Townsend deprivation index (continuous), assessment centre.

^c^ Adjusted for omega-6, age (years; continuous), sex (male, female), race (White, Black, Asian, Others), Townsend deprivation index (continuous), assessment centre.

^d^ Adjusted for omega-6, age (years; continuous), sex (male, female), race (White, Black, Asian, Others), Townsend deprivation index (continuous), assessment centre, BMI (kg/m2; continuous), smoking status (never, previous, current), alcohol intake status (never, previous, current), physical activity (low, moderate, high), and comorbidities (yes, no).

**Table S4.** Associations^a^ of plasma omega-6 PUFAs percentage with all-cause, cancer, and CVD mortality risk in the UK Biobank Study.

| **Omega ratio variable forms** | **Causes of death** | | | | | | | | | | | | | |
| --- | --- | --- | --- | --- | --- | --- | --- | --- | --- | --- | --- | --- | --- | --- |
|  | **All-cause** | | | |  | **Cancer** | | | |  | **Cardiovascular diseases** | | | |
|  | Death # | Model 1^b^ | Model 2^c^ | Model 3^d^ |  | Death # | Model 1^b^ | Model 2^c^ | Model 3^d^ |  | Death # | Model 1^b^ | Model 2^c^ | Model 3^d^ |
|  |  | HR  (95% CI) | HR  (95% CI) | HR  (95% CI) |  |  | HR  (95% CI) | HR  (95% CI) | HR  (95% CI) |  |  | HR  (95% CI) | HR  (95% CI) | HR  (95% CI) |
| Continuous | 6,461 | 0.96 | 0.96 | 0.98 |  | 2,794 | 0.97 | 0.97 | 0.98 |  | 1,668 | 0.94 | 0.94 | 0.99 |
|  |  | (0.95-0.97) | (0.95-0.96) | (0.97-0.99) |  |  | (0.96-0.98) | (0.96-0.98) | (0.97-1.00) |  |  | (0.93-0.96) | (0.93-0.95) | (0.97-1.00) |
| Quintiles (median) | |  |  |  |  |  |  |  |  |  |  |  |  |  |
| 1 (33) | 1,911 | 1.00 | 1.00 | 1.00 |  | 772 | 1.00 | 1.00 | 1.00 |  | 545 | 1.00 | 1.00 | 1.00 |
|  |  | (ref) | (ref) | (ref) |  |  | (ref) | (ref) | (ref) |  |  | (ref) | (ref) | (ref) |
| 2 (37) | 1,426 | 0.82 | 0.82 | 0.90 |  | 628 | 0.86 | 0.86 | 0.91 |  | 363 | 0.76 | 0.76 | 0.87 |
|  |  | (0.76-0.88) | (0.77-0.88) | (0.83-0.97) |  |  | (0.77-0.96) | (0.78-0.96) | (0.80-1.02) |  |  | (0.66-0.87) | (0.67-0.87) | (0.74-1.02) |
| 3 (39) | 1,259 | 0.78 | 0.78 | 0.95 |  | 532 | 0.78 | 0.78 | 0.87 |  | 329 | 0.76 | 0.75 | 1.09 |
|  |  | (0.73-0.84) | (0.73-0.84) | (0.87-1.03) |  |  | (0.70-0.87) | (0.70-0.87) | (0.77-0.99) |  |  | (0.66-0.87) | (0.66-0.87) | (0.92-1.28) |
| 4 (40) | 1,075 | 0.75 | 0.73 | 0.90 |  | 502 | 0.82 | 0.80 | 0.93 |  | 249 | 0.65 | 0.63 | 0.98 |
|  |  | (0.69-0.81) | (0.68-0.79) | (0.83-0.99) |  |  | (0.73-0.92) | (0.71-0.90) | (0.81-1.06) |  |  | (0.56-0.76) | (0.55-0.74) | (0.82-1.17) |
| 5 (42) | 790 | 0.64 | 0.60 | 0.77 |  | 360 | 0.69 | 0.65 | 0.80 |  | 182 | 0.53 | 0.50 | 0.83 |
|  |  | (0.59-0.69) | (0.55-0.65) | (0.70-0.85) |  |  | (0.61-0.78) | (0.57-0.74) | (0.68-0.92) |  |  | (0.45-0.63) | (0.42-0.59) | (0.68-1.02) |
| *P*_trend_ |  | *<0.001* | *<0.001* | *<0.001* |  |  | *<0.001* | *<0.001* | *0.007* |  |  | *<0.001* | *<0.001* | *0.363* |

Abbreviations: CI, confidence interval; HR, hazards ratio; ref, reference.

^a^ From Cox proportional hazards regression.

^b^ Adjusted for age (years; continuous), sex (male, female), race (White, Black, Asian, Others), Townsend deprivation index (continuous), assessment centre.

^c^ Adjusted for omega-3, age (years; continuous), sex (male, female), race (White, Black, Asian, Others), Townsend deprivation index (continuous), assessment centre.

^d^ Adjusted for omega-3, age (years; continuous), sex (male, female), race (White, Black, Asian, Others), Townsend deprivation index (continuous), assessment centre, BMI (kg/m2; continuous), smoking status (never, previous, current), alcohol intake status (never, previous, current), physical activity (low, moderate, high), and comorbidities (yes, no).

**Table S5.** Fully adjusted joint associations^a^ of plasma omega-3 PUFAs percentage and omega-6 PUFAs percentage with all-cause and cause-specific mortality in the UK Biobank Study.

|  | Omega-6% quintiles | | | | | | | | | |
| --- | --- | --- | --- | --- | --- | --- | --- | --- | --- | --- |
|  | All-cause mortality^b^ | | | | | | | | | |
| Omega-3% quintiles | Death/Total | 1  HR (95%CI) | Death/Total | 2  HR (95%CI) | Death/Total | 3  HR (95%CI) | Death/Total | 4  HR (95%CI) | Death/Total | 5  HR (95%CI) |
| 1 | 391/2,830 | 1.00 (Ref)^c^ | 304/2,693 | 0.96 (0.80-1.15) | 289/2,955 | 0.97 (0.81-1.17) | 282/3,444 | 0.93 (0.76-1.12) | 260/5,163 | 0.73 (0.59-0.89) |
| 2 | 370/3,483 | 0.75 (0.64-0.89) | 279/3,092 | 0.80 (0.66-0.97) | 253/3,144 | 0.87 (0.72-1.06) | 220/3,420 | 0.76 (0.61-0.94) | 161/3,946 | 0.64 (0.50-0.81) |
| 3 | 401/3,600 | 0.81 (0.69-0.96) | 265/3,390 | 0.66 (0.54-0.80) | 236/3,362 | 0.70 (0.56-0.86) | 193/3,463 | 0.62 (0.49-0.78) | 151/3,270 | 0.66 (0.82-0.85) |
| 4 | 396/3,598 | 0.79 (0.67-0.94) | 292/3,680 | 0.70 (0.58-0.85) | 222/3,622 | 0.60 (0.48-0.75) | 195/3,428 | 0.69 (0.54-0.87) | 139/2,757 | 0.73 (0.56-0.95) |
| 5 | 353/3,574 | 0.68 (0.57-0.82) | 286/4,230 | 0.55 (0.45-0.68) | 259/4,002 | 0.68 (0.54-0.85) | 185/3,330 | 0.60 (0.47-0.77) | 79/1,949 | 0.48 (0.35-0.67)^d^ |
|  | | | | | | | | | | |
|  | Cancer mortality^b^ | | | | | | | | | |
| 1 | 139/2,830 | 1.00 (Ref)^c^ | 122/2,693 | 1.02 (0.77-1.36) | 98/2,955 | 0.76 (0.55-1.03) | 131/3,444 | 1.03 (0.76-1.40) | 110/5,163 | 0.72 (0.52-1.00) |
| 2 | 132/3,483 | 0.72 (0.55-0.96) | 117/3,092 | 0.87 (0.65-1.17) | 114/3,144 | 0.96 (0.72-1.30) | 106/3,420 | 0.81 (0.59-1.13) | 73/3,946 | 0.64 (0.44-0.93) |
| 3 | 182/3,600 | 0.98 (0.76-1.27) | 118/3,390 | 0.73 (0.54-0.99) | 113/3,362 | 0.74 (0.53-1.03) | 92/3,463 | 0.70 (0.49-0.99) | 76/3,270 | 0.72 (0.49-1.05) |
| 4 | 168/3,598 | 0.93 (0.71-1.21) | 136/3,680 | 0.73 (0.54-1.00) | 95/3,622 | 0.60 (0.43-0.84) | 91/3,428 | 0.68 (0.47-0.99) | 63/2,757 | 0.74 (0.49-1.11) |
| 5 | 151/3,574 | 0.74 (0.56-0.99) | 135/4,230 | 0.63 (0.46-0.87) | 112/4,002 | 0.63 (0.44-0.91) | 82/3,330 | 0.58 (0.39-0.86) | 38/1,949 | 0.53 (0.33-0.86)^d^ |
|  | | | | | | | | | | |
|  | CVD mortality^b^ | | | | | | | | | |
| 1 | 116/2,830 | 1.00 (Ref)^c^ | 82/2,693 | 0.97 (0.69-1.36) | 81/2,955 | 1.15 (0.82-1.61) | 66/3,444 | 0.95 (0.65-1.37) | 68/5,163 | 0.86 (0.59-1.26) |
| 2 | 114/3,483 | 0.73 (0.54-1.00) | 71/3,092 | 0.77 (0.53-1.10) | 61/3,144 | 0.88 (0.61-1.27) | 45/3,420 | 0.79 (0.51-1.22) | 36/3,946 | 0.68 (0.43-1.09) |
| 3 | 100/3,600 | 0.70 (0.51-0.97) | 64/3,390 | 0.52 (0.35-0.78) | 63/3,362 | 0.73 (0.50-1.07) | 46/3,463 | 0.72 (0.46-1.13) | 27/3,270 | 0.56 (0.33-0.95) |
| 4 | 119/3,598 | 0.72 (0.52-0.98) | 66/3,680 | 0.65 (0.44-0.96) | 61/3,622 | 0.66 (0.43-1.01) | 50/3,428 | 0.80 (0.51-1.27) | 27/2,757 | 0.73 (0.42-1.24) |
| 5 | 96/3,574 | 0.63 (0.45-0.89) | 80/4,230 | 0.56 (0.37-0.85) | 63/4,002 | 0.76 (0.49-1.17) | 42/3,330 | 0.75 (0.46-1.21) | 24/1,949 | 0.71 (0.38-1.31)^d^ |

Abbreviations: CI, confidence interval; HR, hazards ratio; ref, reference; omega-3%, omega-3 fatty acids to total fatty acids percentage; omega-6%, omega-6 fatty acids to total fatty acids percentage.

^a^ From Cox proportional hazards regression.

^b^ Adjusted for age (years; continuous), sex (male, female), race (White, Black, Asian, Others), Townsend deprivation index (continuous), assessment centre, BMI (kg/m2; continuous), smoking status (never, previous, current), alcohol intake status (never, previous, current), physical activity (low, moderate, high), and comorbidities (yes, no).

^c^ Reference category: participants who had both low omega-3 fatty acids percentage and omega-6 fatty acids percentage.

^d^ *P*_interaction_ for all-cause, cancer, and CVD mortality 0.04, 0.15, and 0.98, respectively.

**Table S6.** Associations of the dietary PUFAs with all-cause, cancer, and CVD mortality risk in the UK Biobank (n=153,064).

|  | **Dietary Omega-3%^d^** | | |  | **Dietary Omega-6%^e^** | | |  | **Dietary Omega-ratio^f^** | | |
| --- | --- | --- | --- | --- | --- | --- | --- | --- | --- | --- | --- |
| **Cause of death** | All-cause | Cancer | CVD |  | All-cause | Cancer | CVD |  | All-cause | Cancer | CVD |
|  | HR  (95% CI) | HR  (95% CI) | HR  (95% CI) |  | HR  (95% CI) | HR  (95% CI) | HR  (95% CI) |  | HR  (95% CI) | HR  (95% CI) | HR  (95% CI) |
| Continuous | 0.98  (0.96-1.00) | 0.96  (0.93-0.98) | 1.00  (0.96-1.04) |  | 1.00  (0.99-1.00) | 1.00  (0.99-1.01) | 1.00  (0.99-1.01) |  | 1.01  (1.00-1.02) | 1.03  (1.01-1.05) | 0.99  (0.96-1.02) |
| Quintiles^a,b,c^ |  |  |  |  |  |  |  |  |  |  |  |
| 1 | 1.00 (ref) | 1.00 (ref) | 1.00 (ref) |  | 1.00 (ref) | 1.00 (ref) | 1.00 (ref) |  | 1.00 (ref) | 1.00 (ref) | 1.00 (ref) |
| 2 | 0.92  (0.85-0.99) | 0.93  (0.84-1.03) | 0.99  (0.84-1.16) |  | 0.87  (0.81-0.94) | 0.92  (0.83-1.02) | 0.87  (0.74-1.02) |  | 0.99  (0.92-1.07) | 0.99  (0.88-1.10) | 0.96  (0.82-1.13) |
| 3 | 0.96  (0.89-1.04) | 0.95  (0.85-1.06) | 1.09  (0.92-1.29) |  | 0.91  (0.84-0.98) | 0.97  (0.87-1.07) | 0.92  (0.78-1.07) |  | 1.00  (0.92-1.08) | 1.01  (0.90-1.12) | 0.93  (0.79-1.09) |
| 4 | 0.90  (0.82-0.97) | 0.85  (0.75-0.95) | 1.02  (0.85-1.21) |  | 0.90  (0.84-0.98) | 0.93  (0.84-1.04) | 0.98  (0.84-1.15) |  | 1.03  (0.96-1.11) | 1.10  (0.99-1.22) | 0.90  (0.76-1.06) |
| 5 | 0.91  (0.84-0.99) | 0.82  (0.73-0.92) | 1.13  (0.95-1.34) |  | 0.93  (0.86-1.00) | 1.01  (0.90-1.12) | 0.94  (0.80-1.11) |  | 1.05  (0.97-1.13) | 1.15  (1.03-1.28) | 0.96  (0.81-1.12) |
| *P*_trend_ | 0.057 | *<0.001* | *0.128* |  | *0.195* | *0.792* | *0.869* |  | *0.165* | *0.002* | *0.396* |

Abbreviations: CI, confidence interval; HR, hazards ratio; ref, reference.

^a^ Medians of dietary omega-3 percentage in quintiles 1-5 are 1.86, 2.37, 2.77, 3.30, 4.63.

^b^ Medians of dietary omega-6 percentage in quintiles 1-5 are 11.26, 14.01, 16.11, 18.49, 22.57.

^c^ Medians of dietary omega ratio in quintiles 1-5 are 3.49, 5.00, 5.88, 6.75, 8.25.

^d^ From Cox proportional hazards model; adjusted for dietary omega-6 percentage (; continuous), age (years; continuous), sex (male, female), race (White, Black, Asian, Others), Townsend deprivation index (continuous), assessment centre, BMI (kg/m2; continuous), smoking status (never, previous, current), alcohol intake status (never, previous, current), physical activity (low, moderate, high), and comorbidities (yes, no).

^e^ From Cox proportional hazards model; adjusted for dietary omega-3 percentage (; continuous), age (years; continuous), sex (male, female), race (White, Black, Asian, Others), Townsend deprivation index (continuous), assessment centre, BMI (kg/m2; continuous), smoking status (never, previous, current), alcohol intake status (never, previous, current), physical activity (low, moderate, high), and comorbidities (yes, no).

^f^ From Cox proportional hazards model; adjusted for age (years; continuous), sex (male, female), race (White, Black, Asian, Others), Townsend deprivation index (continuous), assessment centre, BMI (kg/m2; continuous), smoking status (never, previous, current), alcohol intake status (never, previous, current), physical activity (low, moderate, high), and comorbidities (yes, no).

**Table S7.** Selected participants serum biochemical markers at baseline across quintiles of the plasma omega-6/omega-3 PUFAs ratio (n=85,425).

|  | **Omega-6/omega-3 ratio quintiles** | | | | |  |
| --- | --- | --- | --- | --- | --- | --- |
| **Biomarker**^a^ | **1** (median = 5.9)  (*n* = 17,085) | **2** (median = 7.6)  (*n* = 17,085) | **3** (median = 9.1)  (*n* = 17,085) | **4** (median = 11.0)  (*n* = 17,085) | **5** (median = 14.8)  (*n* = 17,085) | *P* |
| **Cardiovascular:** |  |  |  |  |  |  |
| CRP (mg/L) | 2.3 (3.8) | 2.5 (4.2) | 2.5 (4.2) | 2.5 (4.2) | 2.7 (4.9) | <0.001^b^ |
| *Missing (n)* | *796* | *775* | *803* | *837* | *809* |  |
| **Cancer:** |  |  |  |  |  |  |
| SHBG (nmol/L) | 53.5 (30.0) | 51.8 (28.8) | 51.3 (27.7) | 51.4 (26.9) | 52.3 (26.2) | <0.001^b^ |
| *Missing (n)* | *2,280* | *2,229* | *2,364* | *2,312* | *2,231* |  |
| TTST (nmol/L) | 5.8 (5.7) | 6.3 (5.8) | 6.7 (6.0) | 7.1 (6.3) | 7.8 (6.5) | <0.001^b^ |
| *Missing (n)* | *2,806* | *2,461* | *2,187* | *2,081* | *1,819* |  |
| E2 (pmol/L) | 445.2 (383.7) | 470.2 (425.8) | 473.9 (503.8) | 457.0 (376.9) | 462.3 (382.1) | <0.001^b^ |
| *Missing (n)* | *14,910* | *14,777* | *14,174* | *13,958* | *13,382* |  |
| IGF-1 (nmol/L) | 21.5 (5.6) | 21.5 (5.5) | 21.7 (5.6) | 21.6 (5.8) | 21.4 (5.9) | <0.001^b^ |
| *Missing (n)* | *864* | *847* | *868* | *886* | *865* |  |

Abbreviations: CRP, C-creative protein; SHBG, sex hormone binding globulin; TTST, testosterone; E2, oestradiol; IGF-1, insulin-like growth factor 1.

^a^ All variables measured at baseline are presented as mean (SD) unless otherwise specified.

^b^ From the ANOVA test for continuous variables.

**Table S8.** Mediation analysis of biomarkers on associations between plasma omega-6/omega-3 ratio and mortality.

|  |  | Path a^a^  (Association between omega-6/omega-3 ratio and biomarker) | Path b^b^  (Association between biomarker and mortality) | Path c^b^  (Association between omega-6/omega-3 ratio and mortality) | Indirect effect | Proportion mediated |
| --- | --- | --- | --- | --- | --- | --- |
| Mortality | Biomarker | Coefficient  (95% CI) | HR Estimate  (95% CI) | HR Estimate  (95% CI) | Estimate  (95% CI) |  |
| All-cause | **Cardiovascular-related:** | | | | | |
|  | CRP | 0.037  (0.029-0.045) | 1.021  (1.016-1.025) | 1.020  (1.014-1.025) | 1.001  (1.001-1.001) | 4.0% |
|  | **Cancer-related:** | | | | | |
|  | SHBG | 0.179  (0.134-0.224) | 1.005  (1.004-1.006) | 1.019  (1.013-1.025) | 1.001  (1.001-1.001) | 5.0% |
|  | TTST | 0.057  (0.051-0.063) | 0.999  (0.989-1.009) | 1.020  (1.015-1.026) | 1.000  (0.999-1.000) | NA |
|  | E2 | -0.066  (-1.565-1.433) | 1.000  (1.000-1.000) | 1.027  (1.013-1.040) | 1.000  (1.000-1.000) | NA |
|  | IGF-1 | -0.084  (-0.094 - -0.074) | 0.992  (0.987-0.998) | 1.020  (1.014-1.025) | 1.001  (1.000-1.001) | 3.4% |
| Cardiovascular | CRP | 0.037  (0.029-0.045) | 1.012  (1.002-1.022) | 1.021  (1.010-1.031) | 1.000  (1.000-1.001) | 2.1% |
| Cancer | SHBG | 0.179  (0.134-0.224) | 1.003  (1.001-1.005) | 1.009  (0.999-1.019) | 1.000  (1.000-1.001) | 5.2% |
|  | TTST | 0.057  (0.051-0.063) | 0.980  (0.965-0.996) | 1.011  (1.001-1.021) | 0.999  (0.998-0.999) | NA |
|  | E2 | -0.066  (-1.565-1.433) | 1.000  (1.000-1.001) | 1.030  (1.009-1.052) | 1.000  (1.000-1.000) | NA |
|  | IGF-1 | -0.084  (-0.094 - -0.074) | 0.999  (0.991-1.007) | 1.012  (1.002-1.022) | 1.000  (0.999-1.000) | 0.8% |

Abbreviations: CRP, C-creative protein; SHBG, sex hormone binding globulin; TTST, testosterone; E2, oestradiol; IGF-1, insulin-like growth factor 1.

^a^ From linear regression model; age (years; continuous), sex (male, female), race (White, Black, Asian, Others), Townsend deprivation index (continuous), assessment centre, BMI (kg/m2; continuous), smoking status (never, previous, current), alcohol intake status (never, previous, current), physical activity (low, moderate, high), and comorbidities (yes, no).

^b^ From Cox proportional hazards model; age (years; continuous), sex (male, female), race (White, Black, Asian, Others), Townsend deprivation index (continuous), assessment centre, BMI (kg/m2; continuous), smoking status (never, previous, current), alcohol intake status (never, previous, current), physical activity (low, moderate, high), and comorbidities (yes, no).

^c^ NA: proportion mediated was not calculated when the point estimate of the direct effect was in an opposite direction to that of the indirect effect.

**Table S9.** Associations^a^ of DHA and LA with all-cause, cancer, and CVD mortality risk in the UK Biobank.

|  | **DHA%**^c^ | | |  | **LA%**^d^ | | |  |
| --- | --- | --- | --- | --- | --- | --- | --- | --- |
| **Cause of death** | All-cause | Cancer | CVD |  | All-cause | Cancer | CVD |  |
|  | HR  (95% CI) | HR  (95% CI) | HR  (95% CI) |  | HR  (95% CI) | HR  (95% CI) | HR  (95% CI) |  |
| Continuous | 0.91  (0.87-0.95) | 0.92  (0.86-0.98) | 0.91  (0.84-1.00) |  | 0.96  (0.96-0.97) | 0.97  (0.95-0.98) | 0.97  (0.96-0.99) |  |
| Quintiles^a,b^ |  |  |  |  |  |  |  |  |
| 1 | 1.00 (ref) | 1.00 (ref) | 1.00 (ref) |  | 1.00 (ref) | 1.00 (ref) | 1.00 (ref) |  |
| 2 | 0.96  (0.88-1.04) | 1.06  (0.93-1.21) | 0.89  (0.76-1.05) |  | 0.86  (0.79-0.93) | 0.86  (0.76-0.97) | 0.84  (0.72-0.98) |  |
| 3 | 0.88  (0.80-0.96) | 0.98  (0.85-1.12) | 0.75  (0.62-0.89) |  | 0.81  (0.74-0.88) | 0.81  (0.71-0.92) | 0.87  (0.74-1.03) |  |
| 4 | 0.83  (0.76-0.91) | 0.90  (0.79-1.04) | 0.81  (0.68-0.97) |  | 0.80  (0.73-0.88) | 0.77  (0.67-0.88) | 0.90  (0.75-1.08) |  |
| 5 | 0.81  (0.74-0.89) | 0.85  (0.74-0.98) | 0.77  (0.64-0.93) |  | 0.66  (0.60-0.73) | 0.69  (0.60-0.81) | 0.67  (0.54-0.83) |  |
| *P*_trend_ | *<0.001* | *0.003* | *0.003* |  | *<0.001* | *<0.001* | *0.001* |  |

Abbreviations: DHA%, docosahexaenoic acid to total fatty acids percentage; LA%, linoleic acid to total fatty acids percentage; CI, confidence interval; HR, hazards ratio; ref, reference.

^a^ Medians of DHA percentage in quintiles 1-5 are 1.24, 1.64, 1.92, 2.25, 2.84.

^b^ Medians of LA percentage in quintiles 1-5 are 24.8, 27.6, 29.4, 31.1, 33.4.

^c^ Adjusted for LA percentage, age (years; continuous), sex (male, female), race (White, Black, Asian, Others), Townsend deprivation index (continuous), assessment centre, BMI (kg/m2; continuous), smoking status (never, previous, current), alcohol intake status (never, previous, current), physical activity (low, moderate, high), and comorbidities (yes, no).

^d^ Adjusted for DHA percentage, age (years; continuous), sex (male, female), race (White, Black, Asian, Others), Townsend deprivation index (continuous), assessment centre, BMI (kg/m2; continuous), smoking status (never, previous, current), alcohol intake status (never, previous, current), physical activity (low, moderate, high), and comorbidities (yes, no).

**Table S10.** Associations^a^ of ratio of omega-6/omega-3 PUFAs with all-cause, cancer, and CVD mortality risk in the UK Biobank, covariates including fish oil supplementation status.

| **Omega ratio variable forms** | **Causes of death** | | | | | | | | | | | |
| --- | --- | --- | --- | --- | --- | --- | --- | --- | --- | --- | --- | --- |
|  | **All-cause** | | |  | **Cancer** | | |  | **Cardiovascular diseases** | | | |
|  | Number of deaths | Partially adjusted associations^b^ | Fully adjusted associations^c^ |  | Number of deaths | Partially adjusted associations^b^ | Fully adjusted associations^c^ |  | Number of deaths | Partially adjusted associations^b^ | | Fully adjusted associations^c^ |
|  |  | HR  (95% CI) | HR  (95% CI) |  |  | HR  (95% CI) | HR  (95% CI) |  |  | HR  (95% CI) | HR  (95% CI) | |
| Continuous | 6,461 | 1.02  (1.02-1.03) | 1.02  (1.01-1.03) |  | 2,794 | 1.02  (1.01-1.03) | 1.01  (1.00-1.02) |  | 1,668 | 1.01  (1.00-1.02) | 1.02  (1.01-1.03) | |
| Quintiles  (median) |  |  |  |  |  |  |  |  |  |  |  | |
| 1 (5.9) | 1,348 | 1.00 (ref) | 1.00 (ref) |  | 593 | 1.00 (ref) | 1.00 (ref) |  | 369 | 1.00 (ref) | 1.00 (ref) | |
| 2 (7.6) | 1,256 | 0.99  (0.91-1.06) | 0.95  (0.87-1.04) |  | 563 | 1.01  (0.90-1.14) | 0.97  (0.85-1.11) |  | 315 | 0.89  (0.76-1.03) | 0.88  (0.74-1.05) | |
| 3 (9.1) | 1,236 | 1.04  (0.96-1.12) | 1.00  (0.91-1.09) |  | 543 | 1.06  (0.94-1.19) | 0.98  (0.85-1.12) |  | 321 | 0.95  (0.81-1.10) | 0.96  (0.80-1.15) | |
| 4 (11.0) | 1,252 | 1.11  (1.03-1.20) | 1.06  (0.97-1.17) |  | 548 | 1.14  (1.01-1.29) | 1.09  (0.95-1.25) |  | 306 | 0.95  (0.81-1.11) | 1.00  (0.84-1.20) | |
| 5 (14.8) | 1,369 | 1.30  (1.20-1.40) | 1.23  (1.12-1.34) |  | 547 | 1.23  (1.09-1.39) | 1.11  (0.97-1.28) |  | 357 | 1.15  (0.99-1.34) | 1.27  (1.07-1.52) | |
| *P*_trend_ |  | *<0.001* | *<0.001* |  |  | *<0.001* | *0.037* |  |  | *0.018* | *<0.001* | |

Abbreviations: CI, confidence interval; HR, hazards ratio; ref, reference.

^a^ From Cox proportional hazards regression.

^b^ Adjusted for age (years; continuous), sex (male, female), race (White, Black, Asian, Others), Townsend deprivation index (continuous), assessment centre, fish oil supplementation.

^c^ Adjusted for age (years; continuous), sex (male, female), race (White, Black, Asian, Others), Townsend deprivation index (continuous), assessment centre, BMI (kg/m2; continuous), smoking status (never, previous, current), alcohol intake status (never, previous, current), physical activity (low, moderate, high), comorbidities (yes, no), and fish oil supplementation.

**Table S11.** Associations^a^ of ratio of omega-6/omega-3 PUFAs with all-cause, cancer, and CVD mortality risk in the UK Biobank Study, with multiple imputation for missing data.

| **Omega ratio variable forms** | **Causes of death** | | | | | | | | | | | |
| --- | --- | --- | --- | --- | --- | --- | --- | --- | --- | --- | --- | --- |
|  | **All-cause** | | |  | **Cancer** | | |  | **Cardiovascular diseases** | | | |
|  | Number of deaths | Partially adjusted associations^b^ | Fully adjusted associations^c^ |  | Number of deaths | Partially adjusted associations^b^ | Fully adjusted associations^c^ |  | Number of deaths | Partially adjusted associations^b^ | | Fully adjusted associations^c^ |
|  |  | HR  (95% CI) | HR  (95% CI) |  |  | HR  (95% CI) | HR  (95% CI) |  |  | HR  (95% CI) | HR  (95% CI) | |
| Continuous | 6,461 | 1.02  (1.02-1.03) | 1.02  (1.01-1.02) |  | 2,794 | 1.02  (1.01-1.03) | 1.02  (1.01-1.02) |  | 1,668 | 1.02  (1.01-1.03) | 1.01  (1.00-1.02) | |
| Quintiles  (median) |  |  |  |  |  |  |  |  |  |  |  | |
| 1 (5.9) | 1,348 | 1.00 (ref) | 1.00 (ref) |  | 593 | 1.00 (ref) | 1.00 (ref) |  | 369 | 1.00 (ref) | 1.00 (ref) | |
| 2 (7.6) | 1,256 | 0.99  (0.92-1.07) | 0.97  (0.90-1.05) |  | 563 | 1.02  (0.91-1.15) | 1.00  (0.89-1.12) |  | 315 | 0.89  (0.77-1.04) | 0.87  (0.74-1.01) | |
| 3 (9.1) | 1,236 | 1.06  (0.98-1.15) | 1.05  (0.97-1.13) |  | 543 | 1.08  (0.96-1.21) | 1.05  (0.94-1.19) |  | 321 | 0.97  (0.84-1.13) | 0.97  (0.83-1.12) | |
| 4 (11.0) | 1,252 | 1.14  (1.05-1.23) | 1.10  (1.02-1.19) |  | 548 | 1.16  (1.03-1.30) | 1.12  (1.00-1.26) |  | 306 | 0.97  (0.84-1.13) | 0.95  (0.82-1.11) | |
| 5 (14.8) | 1,369 | 1.34  (1.24-1.44) | 1.28  (1.19-1.38) |  | 547 | 1.26  (1.12-1.42) | 1.20  (1.07-1.35) |  | 357 | 1.19  (1.03-1.38) | 1.17  (1.01-1.36) | |
| *P*_trend_ |  | *<0.001* | *<0.001* |  |  | *<0.001* | *<0.001* |  |  | *0.004* | *<0.001* | |

Abbreviations: CI, confidence interval; HR, hazards ratio; ref, reference.

^a^ From Cox proportional hazards regression.

^b^ Adjusted for age (years; continuous), sex (male, female), race (White, Black, Asian, Others), Townsend deprivation index (continuous), assessment centre.

^c^ Adjusted for age (years; continuous), sex (male, female), race (White, Black, Asian, Others), Townsend deprivation index (continuous), assessment centre, BMI (kg/m2; continuous), smoking status (never, previous, current), alcohol intake status (never, previous, current), physical activity (low, moderate, high), and comorbidities (yes, no).

**Table S12.** Associations^a^ of ratio of omega-6/omega-3 PUFAs with all-cause, cancer, and CVD mortality risk in the UK Biobank Study, excluding those who died in the first follow-up year.

| **Omega ratio variable forms** | **Causes of death** | | | | | | | | | | | |
| --- | --- | --- | --- | --- | --- | --- | --- | --- | --- | --- | --- | --- |
|  | **All-cause** | | |  | **Cancer** | | |  | **Cardiovascular diseases** | | | |
|  | Number of deaths | Partially adjusted associations^b^ | Fully adjusted associations^c^ |  | Number of deaths | Partially adjusted associations^b^ | Fully adjusted associations^c^ |  | Number of deaths | Partially adjusted associations^b^ | | Fully adjusted associations^c^ |
|  |  | HR  (95% CI) | HR  (95% CI) |  |  | HR  (95% CI) | HR  (95% CI) |  |  | HR  (95% CI) | HR  (95% CI) | |
| Continuous | 6,345 | 1.02  (1.02-1.03) | 1.02  (1.02-1.03) |  | 2,754 | 1.02  (1.01-1.03) | 1.01  (1.00-1.02) |  | 1,620 | 1.02  (1.01-1.03) | 1.02  (1.01-1.03) | |
| Quintiles  (median) |  |  |  |  |  |  |  |  |  |  |  | |
| 1 (5.9) | 1,348 | 1.00 (ref) | 1.00 (ref) |  | 588 | 1.00 (ref) | 1.00 (ref) |  | 360 | 1.00 (ref) | 1.00 (ref) | |
| 2 (7.6) | 1,235 | 1.00  (0.92-1.08) | 0.96  (0.88-1.05) |  | 555 | 1.02  (0.91-1.15) | 0.97  (0.85-1.11) |  | 306 | 0.90  (0.77-1.04) | 0.89  (0.75-1.07) | |
| 3 (9.1) | 1,217 | 1.06  (0.98-1.15) | 1.02  (0.93-1.12) |  | 538 | 1.08  (0.96-1.21) | 1.00  (0.87-1.14) |  | 314 | 0.98  (0.84-1.14) | 0.98  (0.82-1.18) | |
| 4 (11.0) | 1,225 | 1.13  (1.05-1.23) | 1.08  (0.99-1.19) |  | 537 | 1.15  (1.02-1.29) | 1.10  (0.97-1.26) |  | 296 | 0.97  (0.83-1.14) | 1.02  (0.85-1.22) | |
| 5 (14.8) | 1,340 | 1.34  (1.24-1.44) | 1.26  (1.15-1.37) |  | 536 | 1.25  (1.11-1.41) | 1.14  (0.99-1.30) |  | 344 | 1.19  (1.02-1.38) | 1.29  (1.08-1.53) | |
| *P*_trend_ |  | *<0.001* | *<0.001* |  |  | *<0.001* | *0.016* |  |  | *0.005* | *<0.001* | |

Abbreviations: CI, confidence interval; HR, hazards ratio; ref, reference.

^a^ From Cox proportional hazards regression.

^b^ Adjusted for age (years; continuous), sex (male, female), race (White, Black, Asian, Others), Townsend deprivation index (continuous), assessment centre.

^c^ Adjusted for age (years; continuous), sex (male, female), race (White, Black, Asian, Others), Townsend deprivation index (continuous), assessment centre, BMI (kg/m2; continuous), smoking status (never, previous, current), alcohol intake status (never, previous, current), physical activity (low, moderate, high), and comorbidities (yes, no).

**Table S13**. Baseline characteristics of participants with missing exposure information and not included in the study.

| Characteristics^a^ | Not included  (N=416,959) | Included  (N=85,425) |
| --- | --- | --- |
| Age (years) | 56.7 (8.1) | 55.9 (8.2) |
| Gender (male%) | 45.3 | 47.0 |
| Ethnicity(n%) |  |  |
| White | 376,904 (90.9%) | 77,242 (90.9%) |
| Black | 2,347 (0.6%) | 525 (0.6%) |
| Asian | 15,842 (3.8%) | 3,287 (3.9%) |
| Others | 19,504 (4.7%) | 3,955 (4.7%) |
| *Missing (n)* | *2,362* | *416* |
| TDI | -1.3 (3.1) | -1.3 (3.1) |
| *Missing (n)* | *511* | *115* |
| BMI | 27.5 (4.8) | 27.2 (4.7) |
| *Missing (n)* | *2,805* | *302* |
| Smoking status (n%) |  |  |
| Never | 226,722 (54.7%) | 46,736 (55.0%) |
| Previous | 144,202 (34.8%) | 28,811 (33.9%) |
| Current | 43,536 (10.5%) | 9,426 (11.1%) |
| *Missing (n)* | *2499* | *452* |
| Alcohol status (n%) |  |  |
| Never | 18,689 (4.5%) | 3,691 (4.3%) |
| Previous | 15,016 (3.6%) | 3,077 (3.6%) |
| Current | 381,825 (91.9%) | 78,431 (92.1%) |
| *Missing (n)* | *1429* | *226* |
| Physical activity (n%) |  |  |
| Low | 63,283 (19.0%) | 12,907 (18.6%) |
| Moderate | 135,778 (40.8%) | 28,213 (40.7%) |
| High | 133,905 (40.2%) | 28,194 (40.7%) |
| *Missing (n)* | *83,993* | *16,111* |
| Fish oil supplementation (Yes%) |  |  |
|  | 31.5 | 30.7 |
| *Missing (n)* | *5,869* | *327* |

^a^ All variables measured at baseline are presented as mean (SD) except as otherwise specified.
