# Supplementary material for: Higher ratio of plasma omega-6/omega-3 fatty acids is associated with greater risk of all-cause, cancer, and cardiovascular mortality: A population-based cohort study in UK Biobank
Source: eLife. 2024 Apr 5;12:RP90132. doi: 10.7554/eLife.90132 (PMC10997328; doi:10.7554/eLife.90132)
Supplement: Supplementary file 3. [file elife-90132-supp3.docx]

**
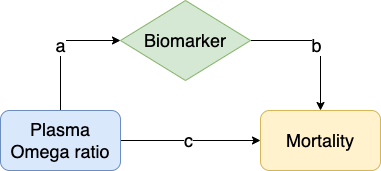
**

**Directed acyclic graph to explain mediation.** The ab arrows represent the indirect effect (i.e., the pathway through the mediator), while the c arrow represents the direct effects (i.e., all pathways other than through the mediator). Sum them up get the total effect which is the overall effect of exposure on outcome in the presence of a mediator.
